# True Temperance

**Published:** 1859-02

**Authors:** 


### TRUE TEMPERANCE.

We do not mean a temperance restricted in its application to spirituous drink, but on the comprehensive scale laid down in the Holy Scriptures, in the injunction to be "Temperate in all things." While it is quite certain that those who begin in their teens to adhere to a rational temperance, may very safely calculate on reaching threescore years and ten, and even fourscore, there is the hope which example and uncontroverted fact give, that even if health is lost at "forty-five," a wise temperance begun and continued from that age, promises the living in comfort and happiness, to double the number of years!

Lewis Cornaro, an Italian nobleman, gifted and rich, yielded to the depravities of his nature, and at the early age of forty-five, found himself a wreck in fortune, fame and health. The physicians whom he consulted, being familiar with his excesses and his reckless character, fortified in their opinion, by the evident fearful inroads which disease had made on his constitution, considered an attempt at restoration so hopeless, that they declined bending their minds to the preparation of a proper prescription, and to save themselves, as they supposed, a useless trouble, they informed him that he was beyond remedial means, and that the best thing he could do would be to reconcile his mind to the inevitable event, and make for it a Christian preparation.

He at once determined that as he had but a short time to live it should be a merry one, and was about casting himself into the maelstrom of a drunken vicious life, but by some unexplained circumstance, a freak possessed him, that at one effort he would cheat death and the doctors, by entering at once upon a life of the most heroic self-denial, and become in all respects a temperate man. So precise was he, that he weighed his food and measured his drink to the end of his life. He regained his health, regained his possessions, resumed his title and his social position, and became a happy-hearted Christian minded gentleman. His whole nature seemed to overflow with kindness to all his race, and on the twelfth of March, fifteen hundred and sixty-five, feeling that he was approaching the termination of his life, and reclining on his cot, the excellent old man exclaimed : "Full with joy and hope I resign myself to thee, most merciful God." He then disposed himself with serenity, and closing his eyes as if about to slumber, gave a gentle sigh, and expired at the age of "ninety-eight years."

